# Comparison of early clinical outcome in carpal tunnel release - mini-open technique with palmar incision vs. endoscopic technique with wrist crease incision-

**DOI:** 10.1186/s12891-023-07151-w

**Published:** 2024-04-01

**Authors:** Ryo Nakamichi, Taichi Saito, Yasunori Shimamura, Masanori Hamada, Keiichiro Nishida, Toshifumi Ozaki

**Affiliations:** 1 Department of Rehabilitation Medicine, Okayama University Hospital, 2-5-1, Shikata-cho, Kitaku, 700-8558 Okayama Japan; 2 Department of Sports Medicine, Dentistry and Pharmaceutical Sciences, Okayama University Graduate School of Medicine, 2-5-1, Shikata-cho, Kitaku, 700-8558 Okayama Japan

**Keywords:** Carpal tunnel syndrome, Mini-open, Endoscopy, Patient-oriented evaluation

## Abstract

**Background:**

The purpose of this study was to examine two techniques for Carpal Tunnel Syndrome, mini-Open Carpal Tunnel Release (mini-OCTR) and Endoscopic Carpal Tunnel Release (ECTR), to compare their therapeutic efficacy.

**Methods:**

Sixteen patients who underwent mini-OCTR in palmar incision and 17 patients who underwent ECTR in the wrist crease incision were included in the study. All patients presented preoperatively and at 1, 3, and 6 months postoperatively and were assessed with the Visual Analogue Scale (VAS) and the Disabilities of Arm, Shoulder and Hand Score (DASH). We also assessed the pain and cosmetic VAS of the entire affected hand or surgical wound, and the patient’s satisfaction with the surgery.

**Results:**

In the objective evaluation, both surgical techniques showed improvement at 6 months postoperatively. The DASH score was significantly lower in the ECTR group (average = 3 months: 13.6, 6 months: 11.9) than in the mini-OCTR group (average = 3 months: 27.3, 6 months: 20.6) at 3 and 6 months postoperatively. Also, the pain VAS score was significantly lower in the ECTR group (average = 17.1) than in the mini-OCTR group (average = 36.6) at 3 months postoperatively. The cosmetic VAS was significantly lower in the ECTR group (average = 1 month: 15.3, 3 months: 12.2, 6 months: 5.41) than in the mini-OCTR group (average = 1 month: 33.3, 3 months: 31.2, 6 months: 24.8) at all time points postoperatively. Patient satisfaction scores tended to be higher in the ECTR group (average = 3.3) compared to the mini-OCTR group (average = 2.7).

**Conclusions:**

ECTR in wrist increase incision resulted in better pain and cosmetic recovery in an early postoperative phase compared with mini-OCTR in palmar incision. Our findings suggest that ECTR is an effective technique for patient satisfaction.

## Backgrounds

Carpal tunnel syndrome is a group of disorders that cause median nerve compression by the increment of the pressure in the carpal tunnel due to various factors [1]. When conservative treatment, such as external fixation, medication, or steroid injection, is ineffective, carpal tunnel release (CTR) is selected as a treatment to decompress the median nerve [1]. There are various surgical techniques for CTR, and Open Carpal Tunnel Release (OCTR) is the traditional technique [2]. OCTR is the procedure to incise the skin approximately 3-3.5 cm on the palm across to the carpal tunnel and open the transverse carpal ligament to confirm median nerve release directly and sufficiently. Although OCTR is a well-established surgical technique, it is often associated with a high incidence of wound scar tenderness, wound appearance, and delayed return to work [3]. Therefore, several surgical techniques have been developed in recent years to address these problems [4, 5].

One technique is mini-OCTR [2]. Mini-OCTR has a shorter skin incision compared to OCTR, with an incision 1.5 cm on the palm and cutting of the proximal end of the transverse carpal ligament through this incision [6, 7]. Several papers reported that this technique is superior to OCTR in terms of fewer wound-related complaints, shorter operative time, and lower incidence of Pillar pain [7–9].

Another technique is Endoscopic Carpal Tunnel Release (ECTR) [2]. ECTR involves arthroscopic incision of the transverse carpal ligament using one or two portal sites. This technique is also reportedly superior to OCTR in patient satisfaction, key pinch strength, return-to-work time, and incidence of wound-related complications [10–12].

Although mini-OCTR and ECTR are reported to be more effective than OCTR, only two reports have compared these two techniques, thus the information is limited. These reports say that median nerve cross-sectional area (CSA) in ECTR patients recover more than that of OCTR patients in the early postoperative phase, but this difference disappears at 6 months postoperatively [13, 14]. These suggest that ECTR is better for recovery than mini-OCTR in a postoperative early phase. Moreover, these two techniques are characterized by minimal incision compared with OCTR, but the information about their cosmetic satisfaction is still unknown. Therefore, the purpose of this study was to compare the results of these two techniques and to verify their therapeutic efficacy including cosmetic satisfaction in the early postoperative phase.

## Materials and methods

### Patients

Sixteen patients who underwent mini-OCTR from January 1, 2018, to March 31, 2021 and seventeen patients who underwent ECTR from April 1, 2021 to March 31, 2022 at three centers were included in the study. The type of surgery was not randomized. All patients were treated by one hand surgeon. The diagnosis of CTS was performed by a preoperative nerve conduction velocity test with the clinical findings, such as sensory impairment or loss along the median nerve territory, as well as characteristic pain, for those who had median neuropathy. Symptomatic carpal tunnel syndrome, such as tumors, amyloidosis, or previous history of fractures around the wrist, was excluded. Additionally, patients with other comorbidities such as insulin-dependent diabetes, polyneuritis, smoking, and rheumatoid arthritis were excluded. Informed consent was obtained from all patients by the hand surgeon. This study was performed with the approval of the Okayama University Hospital (protocol no. 2209-011).

### Surgical techniques

Surgeries were performed using a consistent technique for each group. The details of each surgery are described below.

### Mini-OCTR

**Fig. 1:**
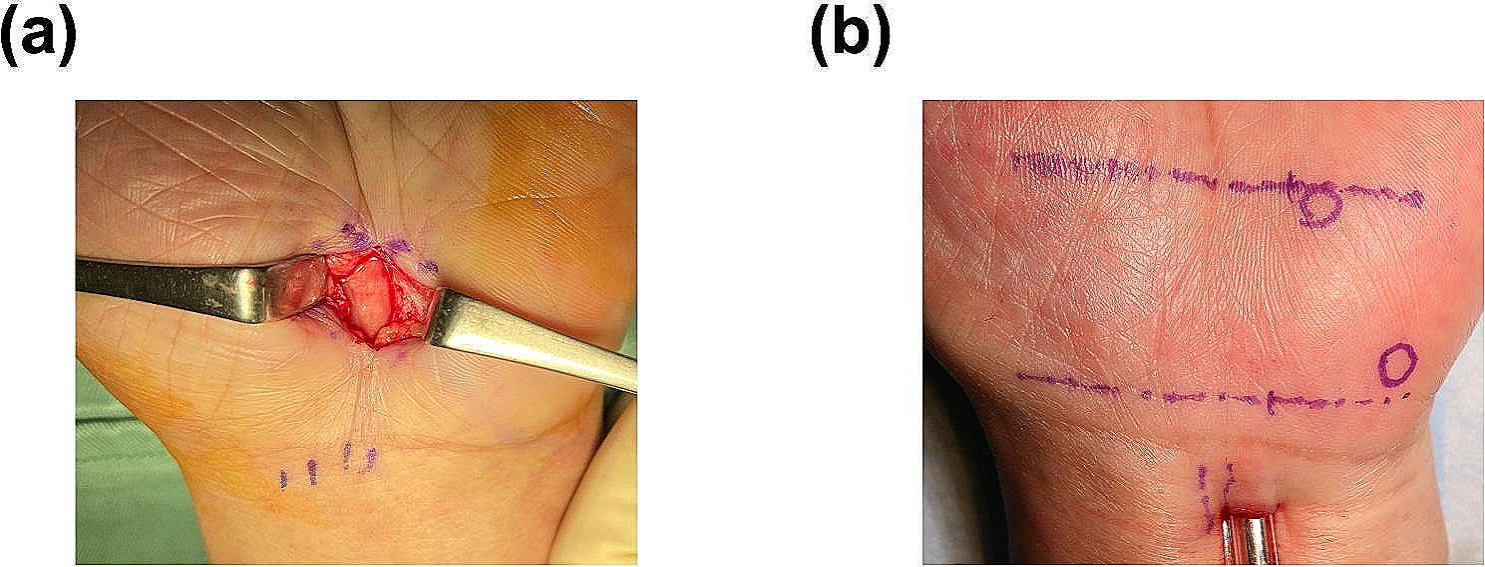
Surgical techniques of Mini-incision Open Carpal Tunnel Release (mini-OCTR) and Endoscopic Carpal Tunnel Release (ECTR). (**a**) A representative view of mini-OCTR. The length of the skin incision is 1.5 cm. (**b**) A representative view of ECTR. The length of the skin incision is 1 cm

Local anesthesia was performed by injection of Xylocaine with 1% epinephrine. No tourniquet was used on the upper arm. The procedure was performed in the supine position; Kaplan’s line was used as the distal end of the skin incision because it has been reported as a safe area that does not damage the palmar artery arch [15–17]. The longitudinal axis in the ulnar border of the third finger and the palmar cutaneous line were used as a guide for skin incision, and approximately 1.5 cm was incised (Fig. 1a). The palmar aponeurosis was incised with a scalpel. After identifying the transverse carpal ligament, a partial incision was performed and the median nerve was identified. The ligament was incised distally and proximally with scissors while a nerve spatula was placed between the median nerve and the ligament to protect it. The incision of the distal end of the ligament was confirmed by direct vision, and the incision of its proximal end was confirmed by inserting the nerve spatula. After washing the surgical area, the skin was closed using interrupted 5 − 0 nylon suture. Bulky dressing with gauze, underwrapping, and elastic bandage were applied after the surgery. From the first postoperative day, the patients were asked to avoid loading the wound, but were allowed to use their hands freely otherwise. Sutures were removed on postoperative days 9–14.

### ECTR

Carpal tunnel block was performed in the operating room with Xylocaine without 1% epinephrine. No tourniquet was used on the upper arm. The procedure was performed in the supine position through a transverse incision 1 cm proximal to the wrist crease and 1 cm from the ulnar margin of the Palmaris longus tendon (Fig. 1b). After blunt incision of the fascia, a tunnel was created below the transverse carpal ligament using a dilator (USE system, Takuto), through which an external tube was inserted (USE system, Takuto). After checking the location of the Median nerve and flexor tendons through an external tube with an endoscope, adjust an external tube to see the transverse ligament directly. A hook (USE system, Takuto) was inserted through the ulnar side of an external tube, and the ligament was incised from the distal end with it. A full incision from the distal to the proximal end was confirmed by observing the soft tissue palmar to the ligament endoscopically. After washing the surgical area, the wound was closed with interrupted 4 − 0 PDS sutures. Bulky dressing with gauze, underwrapping, and elastic bandage were applied after the surgery. From the first postoperative day, the patients were asked to avoid loading the wound, but were allowed to use their hands freely otherwise. The tape was removed on postoperative days 9–14.

### Evaluation

Information on the patient’s gender, age, whether the operated hand was dominant or not, disease duration, and nerve conduction velocity test data were collected from the medical record. Diagnosis and follow-up were performed by one hand surgeon, who recorded changes in symptoms, signs, and adverse events. All patients presented preoperatively and at 1, 3, and 6 months postoperatively and were assessed with the Visual Analogue Scale (VAS) [18] and the Disabilities of Arm, Shoulder and Hand Score (DASH) [19]. We also assessed the pain and cosmetic VAS of the entire affected hand or surgical wound. The patient’s satisfaction with the surgery was assessed with our original questionnaire at 6 months postoperatively. Patients were asked to select one of the five levels of evaluation (5 = Excellent, 4 = Very good, 3 = Good, 2 = Fair, and 1 = Poor). Nerve conduction velocity was also tested at 6 months postoperatively. Complications, such as Pillar pain and recurrence, were evaluated at 1, 3, and 6 months postoperatively. The recurrence was defined as the reappearance of symptoms after a temporary improvement in symptoms, and the recurrence rate was evaluated.

### Statistical analysis

Statistical analyses were performed using Prism 9 (https://www.graphpad.com/). Continuous parameters were presented as mean, 95% confidential interval (CI), and categorical or quantitative data. Descriptive statistics were used for demographic variables; the continuous variables were summarized using SD and/or range via minimum and maximum.

Non-parametric tests were used for data analysis regarding significance. To investigate the differences between the mini-OCTR and ECTR group, Mann–Whitney U tests were utilized. To investigate the differences between four time points in each group, Dunn’s tests were utilized. Fisher’s exact tests were used between two categorical variables. *P*-values (p) below 0.05 were set as statistically significant, and confidence intervals of 95% were computed. A post-hoc power analysis was performed with G*Power 3.1. According to an alpha of 0.05, it was calculated that the sample size could achieve a power of 0.80 based on a two-tailed significance test.

## Results

There were no significant differences in patient characteristics, including gender, age, whether the operated hand was dominant or not, duration of disease, preoperative sensory nerve conduction velocity (SCV), and preoperative distal latency (DL), between the two groups (Table 1). Also, there were no complications in any patients at each time point.

**Table 1:** Patient characteristics

variables	Mini-OCTR group (n = 16)		ECTR group (n = 17)		*P*-value
Sex	4 males, 12 females		7males, 10females		0.465
Dominant	10		7		0.303
	**Average**	**95%CI**	**Average**	**95%CI**	
Age (year-old)	71.6	5.35	66.6	7.20	0.515
Disease duration (months)	15.9	10.1	10.7	3.31	0.598
Preoperative SCV (m/sec)	23.2	6.26	16.4	7.79	0.360
Preoperative DL (msec)	7.42	1.41	7.91	1.30	0.504

SCV = Sensory nerve conduction velocity, DL = Distal latency

**Fig. 2:**
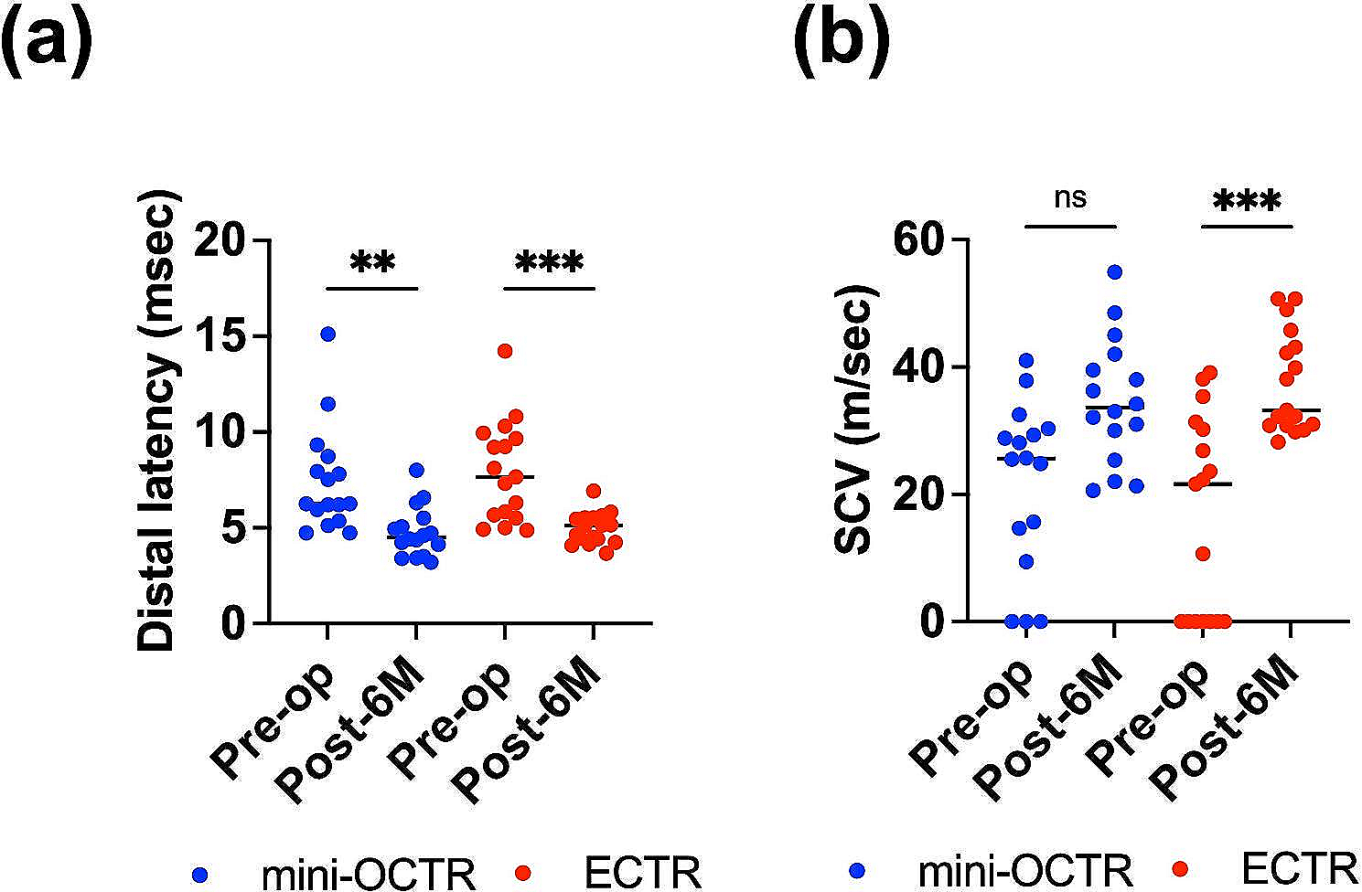
The results of the nerve conduction velocity tests. (**a**) Distal latency of each group preoperatively (Pre-op) and 6 months postoperatively (Post-6 M). Blue dots: mini-OCTR, Red dots: ECTR. (**b**) Sensory nerve conduction velocity (SCV) of each group preoperatively (Pre-op) and 6 months postoperatively (Post-6 M). Blue dots: mini-OCTR, Red dots: ECTR. ** *P* < 0.01, *** *P* < 0.001

Comparing the preoperative with 6-month postoperative nerve conduction velocity, DL showed significant improvement in both groups (Fig. 2a) (Table 2). SCV showed a tendency toward improvement in the mini-OCTR group and a significant improvement in the ECTR group (Fig. 2b) (Table 2). Furthermore, when comparing the results of these two techniques at each time point, there was no difference in DL, SCV, or grip strength ratio between them (Table 2). These results suggest that both techniques are equally effective for decompressing the median nerve using objective evaluation methods.

**Table 2:** The results of all evaluations

		Mini-OCTR (n = 16)		ECTR (n = 17)		*P*-value
		Average	95%CI	Average	95%CI	
DL (msec)	Preop	7.42	2.65	7.92	2.53	0.504
	6 months postop	4.78	1.26	4.97	0.784	0.288
SCV (m/sec)	Preop	21.5	12.9	16.4	15.1	0.36
	6 months postop	34.6	9.57	37.5	7.72	0.428
DASH	Preop	31.7	16.3	28.3	15.7	0.471
	1 month postop	33.3	9.9	26.1	20.4	0.116
	3 months postop	27.3	10.2	13.6	10.4	0.001***
	6 months postop	20.6	9.58	11.9	8.7	0.010*
Pain VAS	Preop	50.7	10.5	43.5	35.3	0.95
	1 month postop	39.4	17.1	29.3	22	0.134
	3 months postop	36.6	17.9	17.1	12	0.001***
	6 months postop	24.6	21.9	17.4	17.7	0.408
Cosmetic VAS	Preop	35	23.7	35.6	33.7	0.95
	1 month postop	33.3	19.2	15.3	15.1	0.011*
	3 months postop	31.2	21.2	12.2	15.2	0.006**
	6 months postop	24.8	25.5	5.41	9.06	0.014*

DL = Distal latency. SCV = Sensory nerve conduction velocity. * *P* < 0.05, ** *P* < 0.01, *** *P* < 0.001

**Fig. 3:**
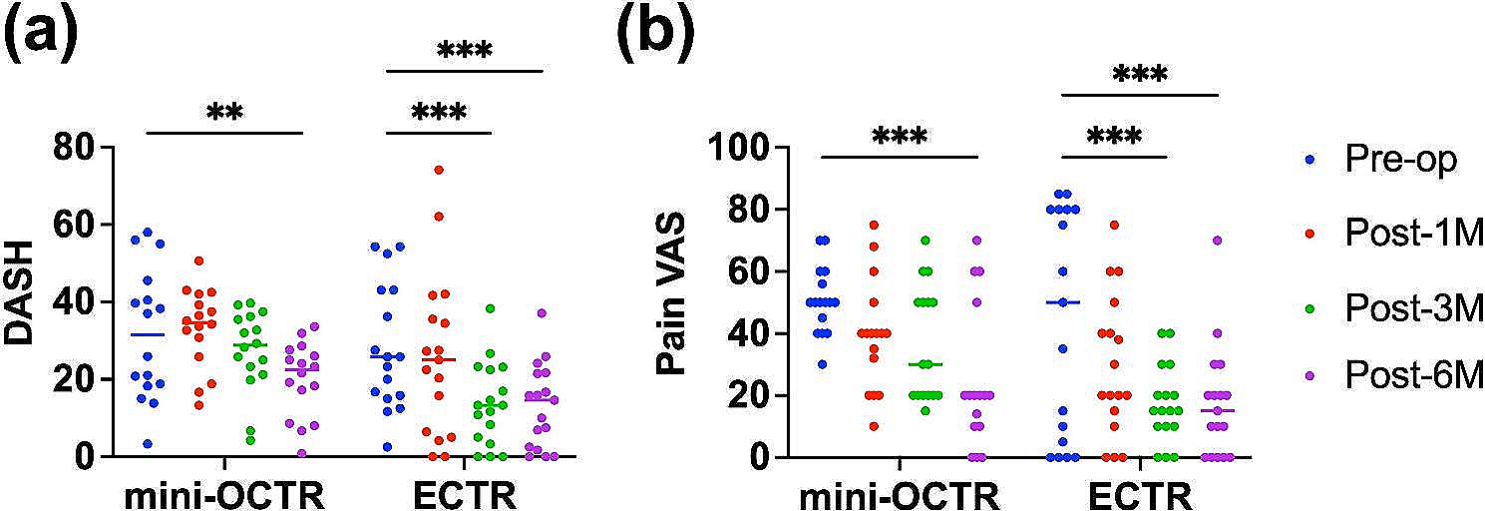
The results of DASH (**a**) and pain VAS (**b**). Blue dots: Preoperatively (Pre-op), Red dots: 1 month postoperatively (Post-1 M), Green dots: 3 months postoperatively (Post-3 M), Purple dots: 6 months postoperatively (Post-6 M).** *P* < 0.01, *** *P* < 0.001

Next, the DASH score in the mini-OCTR group showed significant improvement at 6 months postoperatively compared with preoperatively (Fig. 3a). In contrast, the ECTR group showed significant improvement at 3 months and 6 months postoperatively compared with preoperatively (Fig. 3a) (Table 2). Furthermore, when the DASH score was compared between the two groups at each time point, it was significantly lower in the ECTR group than in the mini-OCTR group at 3 months and 6 months postoperatively (Table 2). These results suggest that ECTR is superior for patient satisfaction with recovery compared with mini-OCTR in the early phase postoperatively (Fig. 3a).

The pain VAS score in the mini-OCTR group showed significant improvement at 6 months postoperatively compared with preoperatively (Fig. 3b) (Table 2). Conversely, the ECTR group showed significant improvement at 3 months and 6 months postoperatively compared with preoperatively (Fig. 3b) (Table 2). Furthermore, when the pain VAS score was compared between the two groups at each time point, it was significantly lower in the ECTR group than in the mini-OCTR group at 3 months postoperatively (Table 2). These results suggest that ECTR is superior for pain relief compared with mini-OCTR in the early phase postoperatively.

**Fig. 4:**
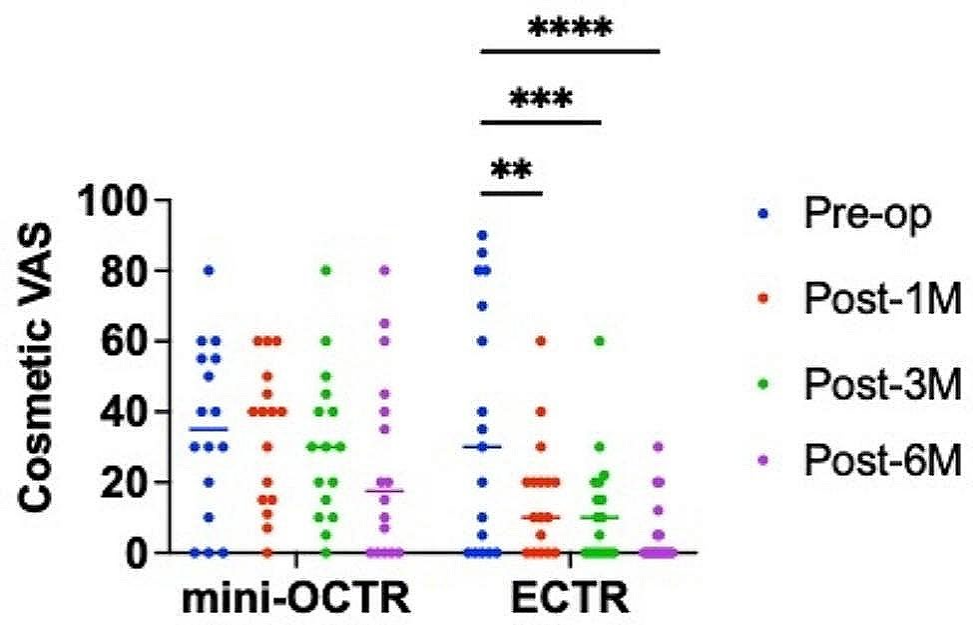
The results of cosmetic VAS. Blue dots: Preoperatively (Pre-op), Red dots: 1 month postoperatively (Post-1 M), Green dots: 3 months postoperatively (Post-3 M), Purple dots: 6 months postoperatively (Post-6 M). ** *P* < 0.01, *** *P* < 0.001, **** *P* < 0.0001

The cosmetic VAS score tended to improve gradually, but the difference was not significant in the mini-OCTR group (Fig. 4) (Table 2). On the contrary, the ECTR group showed significant improvement at 1 month postoperatively compared with preoperatively, which was maintained until the final time point (Fig. 4) (Table 2). Furthermore, when the cosmetic VAS score was compared between the two groups at each time point, it was significantly lower in the ECTR group than in the mini-OCTR group at 1, 3, and 6 months postoperatively (Table 2). These results suggest that ECTR is superior for patients cosmetically compared with mini-OCTR.

**Fig. 5:**
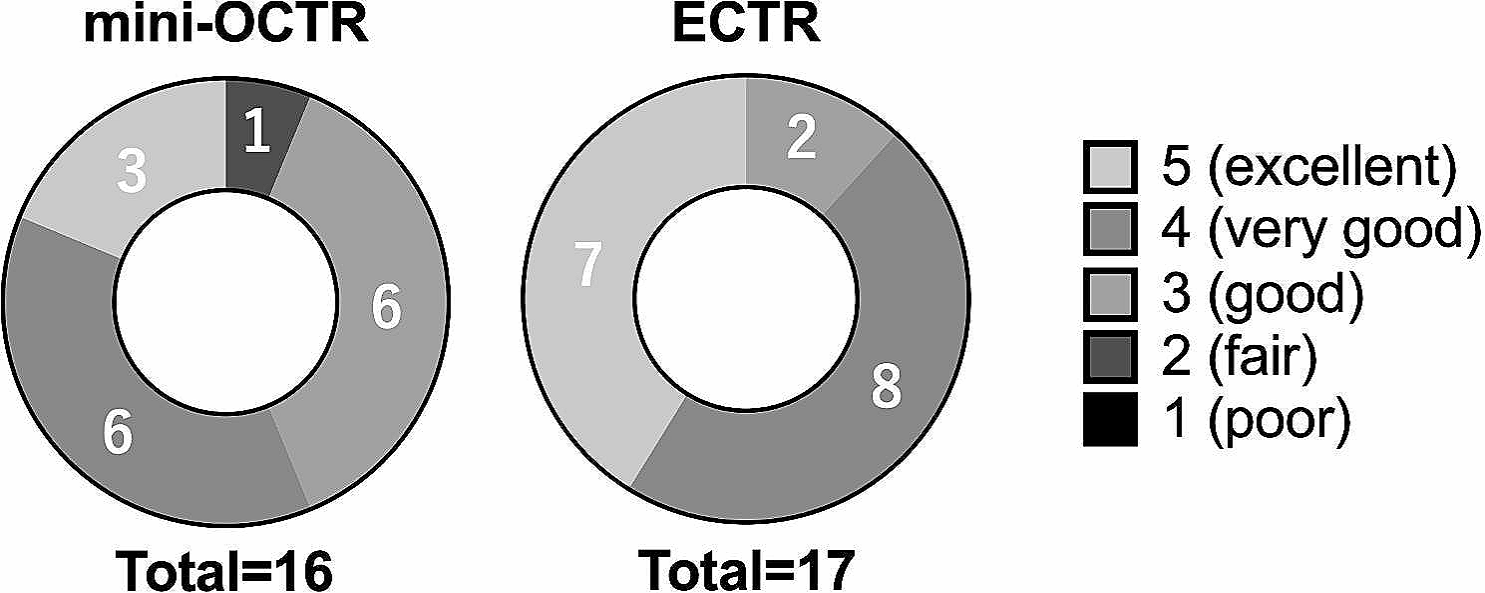
The results of Patient Satisfaction Score. The evaluation consists of five levels (5 = Excellent, 4 = Very good, 3 = Good, 2 = Fair, and 1 = Poor)

At last, patient satisfaction scores tended to be higher in the ECTR group compared to the mini-OCTR group (Fig. 5).

## Discussion

OCTR is a well-established surgical treatment for CTS, however, it has often been associated with a high incidence of wound scar tenderness, wound appearance, and delayed return to work due to wound healing [3]. Both ECTR and mini-OCTR are surgical procedures developed not only to avoid these wound-related problems but also to ensure that the transverse carpal ligament is incised perfectly. In our study, we found that both ECTR and mini-OCTR improved objective evaluations such as nerve conduction velocity and grip strength at 6 months postoperatively. Also, there was no difference between the objective evaluations of each surgical technique, suggesting that both techniques can provide comparable nerve recovery. In patient-oriented evaluations, the DASH score and the pain VAS score were also significantly improved at 6 months after surgery for both surgical techniques. However, surprisingly, ECTR showed earlier recovery than mini-OCTR in both evaluations. A previous report comparing the results of mini-OCTR and that of ECTR showed that there was no difference in the Boston Carpal Tunnel Questionnaire (BCTQ) score and the DASH score at 24 weeks postoperatively, which is consistent with our results at 6 months postoperatively [13]. Furthermore, a study that conducted a surgical questionnaire at 3 months postoperatively for patients who had undergone both surgical techniques for bilateral carpal tunnel syndrome showed that patients preferred ECTR to mini-OCTR [20]. This report shows that “postoperative wound pain” is the most common complaint with mini-OCTR [20]. In our study, the pain VAS score in ECTR patients was improved earlier than that in mini-OCTR patients, suggesting that early pain relief of the wound is one reason that ECTR was preferred to mini-OCTR. Since our one-portal method of ECTR had a 1 cm incision line, and mini-OCTR had a 1.5 cm incision line, ECTR is less invasive than mini-OCTR in appearance, simply. Furthermore, for mini-OCTR, the palmar aponeurosis and soft tissue need to be incised to access the transverse carpal ligament, while the one-portal technique in ECTR allows for quick access to the transverse carpal ligament compared with mini-OCTR, suggesting there is also a difference in surgical invasiveness between the two surgical techniques. Another factor related to postoperative wound pain is that the incision location in ECTR is proximal to the wrist joint, while that in mini-OCTR is on the palm. The skin of the palm is more sensitive than the skin in the area around the wrist joint [21, 22]. Thus, the difference in the characteristics of skin may also affect the wound pain in these techniques.

In our study, the notable finding is that ECTR was superior to mini-OCTR for cosmetic factors. Hand appearance is meaningful because the hand is visible to oneself and the public. This is no exception for patients with hand disorders, being supported by the fact that the hand appearance is already used as a patient-reported outcome for a variety of hand diseases [23–25]. Thus, the surgeon needs to improve both hand function and hand appearance for patient satisfaction in hand surgery. Although this importance is noted in CTS surgery, few reports have evaluated the patient’s cosmetic satisfaction quantitatively [26, 27]. Since the pain VAS score and the DASH score, which are used to assess function in CTS, do not include a cosmetic evaluation, we used the cosmetic VAS to quantify it in this study [28]. Although the mini-OCTR group showed a tendency for improvement in satisfaction with the appearance of the wound over time, the ECTR group showed significant improvement as early as 1 month postoperatively. This may be due to the difference in invasiveness associated with the size of the skin incision, or the difference in ease of recognition due to the difference in wound location, etc. It would be interesting to further study the factors for patient satisfaction in hand appearance.

In this study, there were no complications in any patients. However, complications of both surgical techniques have also been reported, thus the potential disadvantages of mini-OCTR and ECTR should be fully understood [29]. A typical complication of surgical treatment of CTS is injury to the palmar cutaneous branch of the median nerve (PCBMN), which runs in the soft tissue on the palmar side of the transverse carpal ligament [30, 31]. Once injured, the area innervated by the PCBMN can suffer from hypoesthesia or paresthesia, which is called Pillar pain [32]. It has been reported that the incidence of Pillar pain is lower in mini-OCTR than in OCTR [7]. This is due to the difference in the incision length of the palmar site including subcutaneous soft tissues between mini-OCTR and OCTR [30, 31]. In contrast, it has been reported that there is no change in the incidence of Pillar pain when comparing ECTR and OCTR [33–36]. This is because, unlike OCTR, ECTR does not protect the PCBMN from the knife during surgery, which can lead to nerve damage. Especially after cutting the transverse carpal ligament transection one time, there is no protection between the knife and the PCBMN, thus we need to be careful more when the knife is used multiple times from its distal end to the proximal end. Therefore, in ECTR, proficiency in the technique is necessary to avoid this complication [10, 30]. In our study, Pillar pain was not observed in any patients at each time point. It is noted that the ulnar side of the transverse carpal ligament was incised to avoided PCBMN injury in both mini-OCTR and ECTR, and this consideration may have been effective.

One of the limitations of this study is that this is a retrospective case control study ad there was no randomization in the type of surgery performed, suggesting that several biases may not be diminished. Also, we used DASH for evaluation of patient reported outcome, but it may be more beneficial to use a more hand specific index, including Michigan Hand Outcomes Questionnaire (MHQ) and Boston Carpal Tunnel Syndrome Questionnaire (BCTQ) [37, 38]. Additionally, the follow-up period was only 6 months. A previous study showed that the clinical outcomes following surgical release by conventional OCTR improved up to 6 months postoperatively, and this effect was retained 6 years later [39]. In a recent 3-year follow-up comparison of 50 cases of mini-OCTR and 50 cases of conventional OCTR, no recurrence was reported in either group, suggesting that mini-OCTR has a good mid-term outcome as well as OCTR [7]. Since it is important to precisely cut the distal portion of the transverse carpal ligament to prevent recurrence of carpal tunnel release, in our mini-OCTR, a skin incision was made to allow direct visualization of the distal part of the transverse carpal ligament [40]. However, mini-OCTR often makes it difficult to confirm the entire area of the transverse carpal ligament under direct observation, and there is concern about the possibility of leftover incisions [41]. A 7-year follow-up comparison of 53 mini-OCTR cases and 62 conventional OCTR cases showed 9 recurrences in the mini-OCTR group and 5 recurrences in the OCTR group, some of which occurred after more than 5 years, suggesting the need for long-term follow-up to see this complication [41].

In this study, clinical results of mini-OCTR and ECTR were compared, with ECTR showing better improvement in the DASH score and the pain VAS score than mini-OCTR in an early phase postoperatively. The cosmetic VAS score also showed improvement from 1 month postoperatively with ECTR. In conclusion, ECTR is a technique that can be expected to improve symptoms earlier than mini-OCTR.

## Data Availability

The datasets used and/or analysed during the current study available from the corresponding author on reasonable request.
